# Supplementary material for: Analysis of PPARγ Signaling Activity in Psoriasis
Source: Int J Mol Sci. 2021 Aug 10;22(16):8603. doi: 10.3390/ijms22168603 (PMC8395241; doi:10.3390/ijms22168603)
Supplement: Supplementary file 1 [file ijms-22-08603-s001.zip › Supplemental materials_Analysis of PPARg signaling activity in psoriasis/Pathway models/Models images and html files/Model of PPARG signaling in psoriasis/15674.html]

CXCL13


# Protein CXCL13

|  |  |
| --- | --- |
| URN | urn:agi-llid:10563 |
| Total Entities | 0 |
| Connectivity | 1379 |
| Name | CXCL13 |
| Description | C-X-C motif chemokine ligand 13 |
| Notes | B lymphocyte chemoattractant, independently cloned and named Angie, is an antimicrobial peptide and CXC chemokine strongly expressed in the follicles of the spleen, lymph nodes, and Peyer's patches. It preferentially promotes the migration of B lymphocytes (compared to T cells and macrophages), apparently by stimulating calcium influx into, and chemotaxis of, cells expressing Burkitt's lymphoma receptor 1 (BLR-1). It may therefore function in the homing of B lymphocytes to follicles. [provided by RefSeq, Oct 2014] |
| Primary Cell Localization | Extracellular |
| Class | Ligand |

---

|  |  |
| --- | --- |
| Pathway | CXC Chemokine Receptor Signaling |
|  | CD16/CD14 Proinflammatory Monocyte Activation |
|  | Thymic Follicular Hyperplasia |
|  | Proteins Involved in Helicobacter Infections |
|  | Proteins Involved in B-Cell Chronic Lymphocytic Leukemia |
|  | B-Cell Chronic Lymphocytic Leukemia |
|  | Follicular Lymphoma |
|  | Mantle Cell Lymphoma |
|  | Proteins Involved in Marginal Zone Lymphoma |
|  | Proteins Involved in Neuroblastoma |
|  | Proteins Involved in Endometriosis |
|  | Model of PPARG signaling in psoriasis |
|  | 4\_1\_Polymorphisms associated with inflammatory bowel diseases |

---

|  |  |
| --- | --- |
| MedScan ID | 10563 |

---

|  |  |
| --- | --- |
| LocusLink ID | 10563 |
|  | 55985 |
|  | 498335 |

---

|  |  |
| --- | --- |
| Alias | 4631412M08Rik |
|  | ANGIE |
|  | 4631412M08 |
|  | chemokine (C-X-C motif) ligand 13 (B-cell chemoattractant) |
|  | CXC ligand 13 |
|  | CXCL 13 chemokine |
|  | CXCL13 |
|  | chemokine CXC ligand 13 |
|  | CXC chemokine ligand 13 |
|  | Small inducible cytokine B13 |
|  | BCA I |
|  | BLR1L |
|  | B cell-attracting chemokine 1 |
|  | B cell-attracting chemokine I |
|  | C-X-C motif chemokine 13 |
|  | B-cell-homing chemokine (ligand for Burkitt's lymphoma receptor-1) |
|  | LOC115545 |
|  | small inducible cytokine subfamily b (cys-x-cys), member XIII |
|  | C-X-C motif chemokine ligand 13 |
|  | chemokine CXCL13 |
|  | RIKEN cDNA 4631412M08 gene |
|  | CXCL13 chemokine ligand |
|  | SZ13 |
|  | b-cell-homing chemokine |
|  | small inducible cytokine B subfamily (Cys-X-Cys motif), member 13 |
|  | ANGIE2 |
|  | small inducible cytokine subfamily b (cys-x-cys), member 13 |
|  | B lymphocyte chemoattractant |
|  | BCA1 |
|  | small inducible cytokine B subfamily (Cys-X-Cys motif), member 13 (B-cell chemoattractant) |
|  | chemokine (C-X-C motif) ligand 13 |
|  | CXC chemokine BLC |
|  | B-cell chemoattractant 1 |
|  | SCYB13 |
|  | SZ13\_HUMAN |
|  | BLC |
|  | BCA-1 |
|  | B-cell chemoattractant |
|  | B-cell-attracting chemokine 1 |
|  | B-lymphocyte chemoattractant |
|  | small-inducible cytokine B13 |

---

|  |  |
| --- | --- |
| GO ID | 0031735 |
|  | 0048248 |
|  | 0031724 |
|  | 0008009 |
|  | 0017134 |
|  | 0008201 |
|  | 0046982 |
|  | 0048018 |
|  | 0035754 |
|  | 0007186 |
|  | 0090630 |
|  | 0061844 |
|  | 0007166 |
|  | 0007267 |
|  | 0071222 |
|  | 0070098 |
|  | 0002544 |
|  | 0042742 |
|  | 0035768 |
|  | 0002467 |
|  | 0006955 |
|  | 0006954 |
|  | 0030595 |
|  | 0002518 |
|  | 2000545 |
|  | 0030593 |
|  | 0010820 |
|  | 0033634 |
|  | 0007204 |
|  | 0033625 |
|  | 0045765 |
|  | 0002920 |
|  | 0005623 |
|  | 0005576 |
|  | 0005615 |
|  | 0005125 |
|  | 0035769 |
|  | 0006935 |
|  | 0006952 |
|  | 0048535 |
|  | 0042127 |
|  | 0032496 |
|  | 0002690 |
|  | 0045236 |
|  | 0005829 |
|  | 0032861 |
|  | 2000273 |

---

|  |  |
| --- | --- |
| KEGG ID | hsa:10563 |
|  | mmu:55985 |

---

|  |  |
| --- | --- |
| Organism | Homo sapiens {Organism urn:agi-taxid:9606} |
|  | Mus musculus {Organism urn:agi-taxid:10090} |
|  | Rattus norvegicus {Organism urn:agi-taxid:10116} |
|  | Homo sapiens |
|  | Mus musculus |
|  | Rattus norvegicus |

---

|  |  |
| --- | --- |
| Mouse chromosome position | 5 |

---

|  |  |
| --- | --- |
| OMIM ID | 605149 |

---

|  |  |
| --- | --- |
| Rat chromosome position | 14p22 |

---

|  |  |
| --- | --- |
| Hugo ID | 10639 |
|  | HGNC:10639 |

---

|  |  |
| --- | --- |
| Human chromosome position | 4q21.1 |
|  | 4q21 |

---

|  |  |
| --- | --- |
| Swiss-Prot Accession | O43927 |
|  | Q53X90 |
|  | O43927.1 |
|  | O55038 |
|  | Q3U1E8 |
|  | O55038.1 |
|  | Q5I0J6 |
|  | F7F7W7 |

---

|  |  |
| --- | --- |
| GenBank ID | NC\_000004 |
|  | NM\_006419 |
|  | NP\_006410 |
|  | XM\_006714063 |
|  | XP\_006714126 |
|  | AC092674 |
|  | AC093758 |
|  | AAY40955 |
|  | CH471057 |
|  | EAX05816 |
|  | EAX05817 |
|  | EF064743 |
|  | ABK41926 |
|  | JC535575 |
|  | CEF39385 |
|  | AF029894 |
|  | AAC17980 |
|  | AF044197 |
|  | AAC14402 |
|  | AJ002211 |
|  | CAA05250 |
|  | BC012589 |
|  | AAH12589 |
|  | BI836728 |
|  | CA418514 |
|  | CR407624 |
|  | CAG28552 |
|  | O43927 |
|  | NC\_000071 |
|  | NM\_018866 |
|  | NP\_061354 |
|  | AC146611 |
|  | CH466529 |
|  | EDL20365 |
|  | JC535577 |
|  | CEF39386 |
|  | AF030636 |
|  | AAC34603 |
|  | AF044196 |
|  | AAC14401 |
|  | AK156025 |
|  | BAE33550 |
|  | AK160241 |
|  | AV229497 |
|  | BB612247 |
|  | BC012965 |
|  | AAH12965 |
|  | O55038 |
|  | NC\_005113 |
|  | XM\_006250710 |
|  | XP\_006250772 |
|  | NM\_001017496 |
|  | NP\_001017496 |
|  | AC\_000082 |
|  | AABR07014417 |
|  | AAHX01078247 |
|  | CH474022 |
|  | EDL99643 |
|  | BC088260 |
|  | AAH88260 |
|  | FQ234803 |
|  | NC\_018915 |
|  | AMYH02009524 |
|  | AMYH02009525 |
|  | HQ448702 |
|  | ADQ33180 |
|  | AC\_000027 |
|  | AAHY01047018 |
|  | AC\_000136 |
|  | ABBA01063371 |
|  | ABBA01063372 |
|  | ABBA01063373 |

---

|  |  |
| --- | --- |
| Swiss-Prot ID | CXL13\_HUMAN |
|  | CXL13\_MOUSE |

---

|  |  |
| --- | --- |
| Cell Localization | Secreted |

---

|  |  |
| --- | --- |
| Ensembl ID | ENSG00000156234 |
|  | ENSP00000286758.4 |
|  | ENST00000286758.4 |
|  | ENSMUSG00000023078 |
|  | ENSMUSP00000023840.5 |
|  | ENSMUST00000023840.6 |
|  | ENSRNOG00000024899 |
|  | ENSRNOP00000034304.4 |
|  | ENSRNOT00000039383.4 |
|  | ENSP00000286758 |
|  | ENST00000286758 |
|  | ENSMUSP00000023840 |
|  | ENSMUST00000023840 |
|  | ENSRNOP00000034304 |
|  | ENSRNOT00000039383 |

---

|  |  |
| --- | --- |
| MGI ID | MGI:1888499 |

---

|  |  |
| --- | --- |
| RGD ID | 1564256 |

---

|  |  |
| --- | --- |
| Unigene ID | Hs.100431 |
|  | Mm.10116 |
|  | Rn.6917 |

---

|  |  |
| --- | --- |
| Homologene ID | 48431 |

---

|  |  |
| --- | --- |
| Shape | Rhomb |

---
